# The cost-effectiveness of different generations ceramic-on-ceramic implants in primary total hip arthroplasty: a matched population-based study

**DOI:** 10.1186/s12913-025-13792-5

**Published:** 2025-12-09

**Authors:** Yu-Han Huang, Ta-Wei Tai, Shu-Han Hsu, Daphne I. Ling, Jung-Der Wang, Li-Jung Elizabeth Ku

**Affiliations:** 1 Department of Rehabilitation, E-DA Hospital, Kaohsiung, Taiwan; 2 Department of Occupational Therapy, I-Shou University, Kaohsiung, Taiwan; 3 Department of Orthopedics, National Cheng Kung University Hospital, College of Medicine, National Cheng Kung University, Tainan, Taiwan; 4 Skeleton Materials and Biocompatibility Core Lab, Research Center of Clinical Medicine, National Cheng Kung University Hospital, College of Medicine, National Cheng Kung University, Tainan, Taiwan; 5 Department of Public Health, College of Medicine, National Cheng Kung University, Tainan, Taiwan; 6 Division of Occupational and Environmental Medicine, National Cheng Kung University Hospital, College of Medicine, National Cheng Kung University, Tainan, Taiwan

**Keywords:** Ceramic, Implant, Revision, Cost-effectiveness, Total hip arthroplasty

## Abstract

**Background:**

Ceramic-on-ceramic (CoCs) implants offer potential durability benefits in total hip arthroplasty (THA) but require notably high out-of-pocket copayments in Taiwan. This study assessed the cost-effectiveness of third- (3rd-CoCs) and fourth-generation CoC implants (4th-CoCs) compared with fully covered metal-on-polyethylene (MoPs) from a payer’s perspective.

**Methods:**

Using Taiwan’s National Health Insurance (NHI) claims data (2009–2019), we identified osteoarthritis patients aged ≥ 50 years undergoing their first primary THA. We applied both exact matching and propensity score matching between patients who received 3rd- and 4th-CoCs and those who received MoPs. Cox regression and generalized linear models were used to assess clinical outcomes and total healthcare costs, including NHI payments and implant copayments. Incremental cost‒effectiveness ratios (ICERs) were calculated via 1,000 bootstrap iterations.

**Results:**

This 10-year retrospective cohort included 15,233 patients (10,158 MoPs; 1,565 3rd-CoCs; 3,504 4th-CoCs), with median follow-up durations of 6.2 years for 3rd-CoCs and 3.3 years for 4th-CoCs. Compared with MoPs, 3rd-CoCs had lower adjusted hazard ratios (HR) for revision (HR 0.53, 95% CI 0.34–0.85) and postoperative complications (HR 0.69, 95% CI 0.49–0.99), with ICERs of US$704 per 1% gain in revision-free survival and US$794 per 1% gain in postoperative complication-free survival, respectively. 4th-CoCs reduced 90-day medical complications (HR 0.29, 95% CI 0.15–0.54) but had higher ICERs of US$2,947 per 1% gain in medical complication-free survival.

**Conclusions:**

Nationwide data suggests that 3rd-CoCs appear to be more cost-effective than MoPs. In contrast, 4th-CoCs demonstrated limited short-term value and uncertain cost-effectiveness, warranting future long-term evaluation.

**Supplementary Information:**

The online version contains supplementary material available at 10.1186/s12913-025-13792-5.

## Background

Hip osteoarthritis (OA) is a common condition in individuals over 50 years old, characterized by irregularities in the hip joint surface, deformities, pain, and functional loss, leading to limitations in daily activities and mobility. In developed countries, severe OA is one of the leading indications of total hip arthroplasty (THA) [1]. Among the bearing surface options, ceramic-on-ceramic (CoC) and metal-on-polyethylene (MoP) are commonly used [2]. CoC implants are scratch-resistant and chemically inert [3, 4], making them a preferred choice for younger and more active patients due to their excellent early and midterm prosthetic survival rates [5]. However, CoCs have their own complications, such as ceramic-related noises and ceramic component fractures [6]. Technological advances in ceramic materials have led to the development of third-generation CoC implants (3rd-CoCs) and fourth-generation CoC implants (4th-CoCs). The 3rd-CoCs utilize alumina ceramics, while the 4th-CoCs incorporate zirconia into the alumina matrix ceramics, increasing friction resistance and improving bio-lubrication stability [7, 8]. A register study analyzing over 223,000 CoC THA cases in the UK found ceramic head fracture rates of 0.009% for 4th-CoCs versus 0.119% for 3rd-CoCs, demonstrating significantly lower fracture risk with the newer material [9]. Furthermore, a longitudinal study in Korean supported 4th-CoCs reported a lower incidence of head fractures, although liner fracture rates were similar [6].

Despite their clinical potential, the affordability of CoCs varies across countries due to differences in health insurance reimbursement and copayment policies [2, 10]. For instance, CoCs are the most common THA implant in Korea, driven by the medical delivery system and payment plan [2], whereas fewer than 10% of Medicare beneficiaries in the US receive CoCs, presumably due to factors such as implant cost, hospital contracts, and surgeon preferences [10]. In Taiwan, the National Health Insurance (NHI) program—which covers more than 99% of the population—fully covers MoPs but requires copayments for CoCs, which cost 1.2 to 2.7 times more [11]. Although CoCs involve out-of-pocket expenses (OOP) for patients, our literature search found only two studies that examined the cost-effectiveness of CoCs, and they reached different conclusions [12, 13]. One expected-value decision-analysis model—implemented using a Markov framework—estimated that, among 50-year-old patients, an incremental cost of US$2,000 would be cost-saving if it reduced the 20-year implant failure probability by at least 18.7% compared with MoPs, and cost-effective if the failure probability decreased by 3.8% under a willingness-to-pay threshold (WTP) of US$50,000 per quality-adjusted life years (QALYs) gained [12]. However, a more recent Markov model study that comparing 24 THA implant combinations found that CoCs were four times more expensive than traditional MoPs, with uncertain reductions in revision risk, thereby limiting their cost-effectiveness [13]. In addition, another study suggested that higher OOP spending in hip replacement was associated with better care quality, such as shorter length of stay (LoS), although that analysis did not distinguish by implant type [14].

While simulation-based models offer theoretical insight, there remains a lack of real-world data evaluating whether the higher copayments associated with CoCs translate into improved outcomes or economic value. To address this gap, this study uses Taiwan’s nationwide claims data to examine the clinical outcomes, healthcare costs, and incremental cost-effectiveness ratios (ICER) of 3rd- and 4th-generation CoC implants, with MoP implants serving as a reference. Our findings aim to inform both patients and third-party payers regarding the actual clinical and economic returns of copay-based implant selection.

## Methods

### Study setting

This retrospective cohort study used Taiwan’s NHI claims data from patients from 2009 to 2019 and was conducted from the payer’s perspective. Our study was approved by the Institutional Review Board of National Cheng Kung University Hospital (IRB No. B-ER-109-508), which granted a waiver of informed consent because the analysis used only de-identified secondary data. The data were obtained from the Health and Welfare Data Science Center (HWDC), Ministry of Health and Welfare, Taiwan. These data are legally restricted and cannot be made publicly available.

Supplementary Table 1 shows a complete list of International Classification of Diseases, Ninth or Tenth Revision, Clinical Modification/Procedure Coding System (ICD-9-CM/PCS or ICD-10-CM/PCS) codes, and NHI reimbursement codes that were used to identify study patients and outcomes. Patients aged 50 years or older who underwent unilateral primary THA due to OA from January 1, 2010, until December 31, 2018, were included and followed for at least one year (Supplementary Fig. 2). Patients who were diagnosed with autoimmune arthritis, ankylosing spondylitis, or avascular necrosis were excluded because of differences in pathogenesis. Additionally, patients with a history of cancer were excluded to minimize interference, as the variety of cancer types and staging complexity might affect both the choice of implant and the prognosis. Finally, patients who underwent bilateral THA were excluded because of the statistical assumption of independent observations. In addition, patients with missing age or sex variables (*n* = 39) were excluded.

### Creating comparable groups via exact and propensity score matching

A one-to-one, two-step hybrid matching strategy was employed, to ensure strict covariate balance on key clinical variables while adjusting for additional confounders and to create comparable groups. First, exact matching was performed on age (in 5-year intervals), sex, procedure year, and five major diseases [15, 16]. Subsequently, greedy nearest-neighbor matching without replacement was applied within each exact-matched stratum using a caliper width of 0.2 standard deviations of the logit of the propensity score, which is a widely recommended practice to minimize bias and mean squared error [17]. By matching procedure years, we ensured that all matched pairs had identical time horizons. The five major diseases—acute myocardial infarction, stroke, chronic obstructive pulmonary disease, end-stage renal disease, and cirrhosis—were included in the exact matching because we assumed that patients diagnosed with these conditions within 365 days prior to THA were more likely to opt for MoPs owing to their lower life expectancy [18, 19]. This approach allowed us to retain covariate balance on key variables, while further adjusting for patient, surgeon, and institutional characteristics. Unmatched individuals were not included to ensure the comparability of matched pairs.

Propensity scores adjusted for additional patient factors (e.g., diabetes, chronic kidney disease, and socioeconomic status), surgeon factors, and healthcare provider preferences. Socioeconomic status was categorized into four groups according to NHI-insured income: low-income household, occupational union, and salary below and above US$1,078. In addition, surgeons with greater surgical volumes and more experience are more likely to prevent adverse clinical outcomes [20]. The surgeon’s seniority and surgical volume (number of primary/revision THAs within 365 days before the procedure) were considered, with >75% as a high-volume cutoff point [20, 21]. Given Taiwan’s implementation of a bundled payment system for THA since 2010 [22], hospital factors, including location [23], facility levels, and ownership, were controlled for in the propensity score model.

### Defining effectiveness outcomes in THA

The outcomes were the event-free survival rates and hazard ratios (HR) of revision, postoperative complications, and medical complications within 90 days following primary THA. These outcomes directly capture implant survival and complication risks, which are common endpoints in THA procedures. A ‘revision’ refers to the replacement of any part of an implant. The postoperative complications assessed included prosthetic joint infection, periprosthetic fracture, dislocation, and all-cause revision. Additionally, we evaluated 90-day medical complications such as pulmonary embolism, pneumonia, deep vein thrombosis, and sepsis.

### Calculating the NHI and OOP costs

The healthcare costs assessed in the current study included both claim costs paid by NHI and implant copayment paid by the patient. The NHI costs were accumulated for every patient from the initial hospitalization for THA until death or the end of this study, encompassing both in- and outpatient claims. Implant OOP costs were obtained from the public records obtained from the Ministry of Health and Welfare in Taiwan starting in 2015. For procedures performed between 2010 and 2014, direct OOP implant cost data were unavailable. To address this, we applied a multi-step hierarchical imputation strategy. This involved estimating OOP costs by multiplying each patient’s inpatient NHI-reimbursed costs by the average cost-sharing ratios of the implant type. These ratios were calculated based on 2015–2018 data and stratified by hospital level and geographic region to account for variation in reimbursement practices. For example, if a specific implant cost constituted on average 37% of total reimbursed inpatient costs between 2015 and 2018, we assumed a similar cost-sharing pattern applied for the period 2010–2014 and used this proportion to estimate the missing OOP values. As Taiwan’s NHI is operated under a global budget (GB) payment system, we adjusted total healthcare expenditures using GB values, consistent with previous literature [24], to account for annual and sectoral fluctuations in point values (ranging between 0.9 and 1). The total healthcare costs in the NTD were converted to USD using the average exchange rate for the final year of our study (2019), which was 30.898. Because the analysis was based solely on historical costs, discounting of future costs was not applicable.

### Analytical methods for matched pairs and cost estimation

The demographic characteristics of the matched pairs are reported as the means and standard deviations for continuous variables and percentages for categorical variables. We assessed the distribution of the matched covariate imbalance using the standardized mean difference (SMD), considering an SMD greater than 0.1 as an indicator of imbalance. Additionally, we employed Cox regression models, adjusting for patient, surgeon, healthcare provider preference, and other confounding factors, including the use of bone cement [25], the use of modular femoral stems [26], the Elixhauser comorbidity index, and the LoS during primary THA. We examined the proportionality of the hazards assumption by examining covariates and the log of the observation time. Furthermore, we utilized generalized linear models (GLM) with a gamma distribution and log-link function to estimate healthcare costs, adjusting for covariates included in the Cox regressions and follow-up duration.

### Constructing robust ICERs for CoCs and MoPs

To create the cost-effectiveness profile, we calculated the mean differences in event-free survival rates and healthcare costs separately between two CoCs and MoPs. Event-free survival rates were predicted via a Cox regression model, and healthcare costs were calculated on the basis of GLM predictions. Both incremental event-free survival rates and incremental healthcare costs were obtained by subtracting the event-free survival rate of MoPs from that of 3rd- or 4th-CoCs. For effectiveness measures, the time frame varied by outcomes for revision-free and postoperative complications-free survival. These outcomes were assessed over the full follow-up period from 2010 to 2019, while medical complications-free survival was evaluated within 90 days following THA procedure. Because of lacking health utility measures in the claims database, we adopted event-free survival as a practical and clinically meaningful proxy for QALYs.

The ICERs were calculated by dividing incremental healthcare costs by incremental event-free survival rates. To ensure the robustness of ICERs, we generated 1,000 bootstraps to predict event-free survival rates and individual-level healthcare costs, and to calculate 95% confidence intervals (CI) of the estimated ICER. The ICER estimates were visually inspected using scatterplots, which demonstrated a tight clustering around the mean, indicating consistent and stable outcomes.

All P values and 95% CIs were derived from two-tailed tests, with statistical significance set at *P* < 0.05. The data analysis was conducted via SAS 9.4 statistical software and R packages.

## Results

### Demographic and clinical characteristics

Of the 15,227 participants who underwent unilateral primary THA, 10,158 (66.7%) received MoPs, 1,565 (10.3%) received 3rd-CoCs, and 3,504 (23.0%) received 4th-CoCs. After matching, the baseline characteristics were balanced between groups, with all SMDs below 0.1. The mean age of 65 years, and 71% were women (Table 1**)**. Supplementary Fig. 1 illustrates trends in implant uptake and follow-up over the study period: 3rd-CoCs were already in use in Taiwan prior to 2010, whereas 4th-CoCs became available in 2011.

**Table 1:** Frequency distributions of demographic and clinical characteristics of matched participants who underwent total hip arthroplasty from 2010–2018 in Taiwan

	3rd-CoCs group (1,498 pairs)			4th-CoCs group (2,985 pairs)		
	MoPs (%)	3rd-CoCs (%)	SMD^a^	MoPs (%)	4th-CoCs (%)	SMD^a^
Median follow-up (year, Q1–Q3)	6.2 (4.1–8.0)		-	3.3 (2.1–5.0)		-
Age (Mean ± SD)	65.1 ± 8.4	65.0 ± 8.3	0.00	65.2 ± 8.0	65.0 ± 7.9	0.02
Woman	71.0	71.0	0.00	71.3	71.3	0.00
Insured amount/status						
Low-income household	16.5	17.4	0.00	16.0	15.9	0.00
Occupational union	51.6	50.0	0.00	49.9	44.3	0.00
Salary ≤ 1078/month ^b^	21.2	18.0	0.01	23.4	21.5	0.00
Salary > 1078/month ^b^	10.7	14.6	0.01	10.6	18.3	0.01
Hospital ownership						
Government-owned	47.9	47.9	0.00	31.5	30.5	0.00
Not-for-profit hospitals	43.7	43.0	0.00	48.9	49.9	0.00
Private hospitals	8.4	9.1	0.00	19.6	19.5	0.00
Healthcare facility level						
Medical center	39.3	41.6	0.00	46.2	49.1	0.00
Regional hospital	31.1	30.3	0.00	33.8	31.0	0.01
District hospital	29.6	28.1	0.00	20.0	20.0	0.00
Comorbidities						
Diabetes	14.6	14.3	0.00	16.1	15.8	0.00
Chronic kidney disease	2.0	2.5	0.00	02.0	02.1	0.00
Years of specialty practice ≤ 23 ^c^	84.0	81.6	0.00	67.5	62.1	0.01
Surgeon’s surgical volume ≤ 59 in the year before surgery ^d^	78.9	79.5	0.00	76.5	71.8	0.01
Copay of implants (median, Q1–Q3)	1,348.2 (1,213.3–1,554.4)		-	3,196.3 (1,826.4–3,421.4)		-

MoPs indicate metal-on-polyethylene implants; CoCs, ceramic-on-ceramic implants

a. * Standardized mean difference (SMD) >0.1 index imbalance

b. The 50^th^ percentile of salary is US$1,078

c. The 75^th^ percentile of years of Specialty Practice is 23 years

d. The 75^th^ percentile of surgeon‘s surgical volume in the year before surgery is 59

### Clinical effectiveness outcomes

**Fig. 1:**
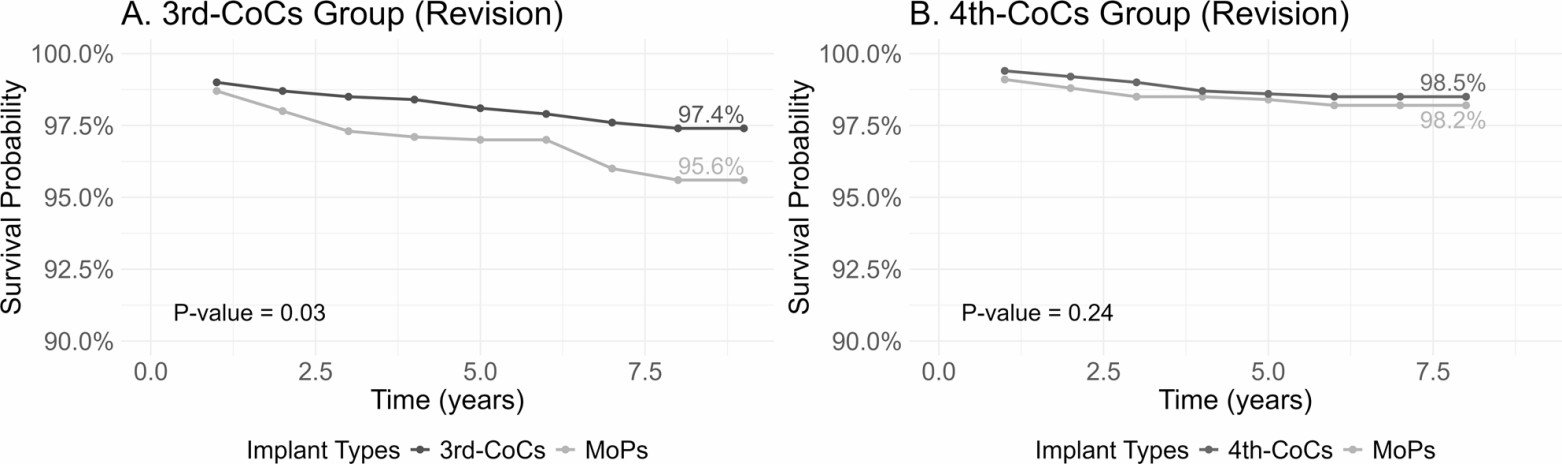
Revision-free survival rates of two generations of CoCs and matched MoPs. Notes: MoPs indicate metal-on-polyethylene implants; CoCs, ceramic-on-ceramic implants. The y-axis started at 90% to provide a detailed view of the high survival probability range

Over a median follow-up period of 6.2 years, the revision-free survival rates of 3rd-CoCs and matched MoPs were 97.4% and 95.6%, respectively (*P* = 0.03; Fig. 1). Additionally, 3rd-CoCs were associated with a lower risk of revision (HR 0.53, 95% CI 0.34–0.85; Table 2). Figure 1 also show that over a median follow-up period of 3.3 years, the revision-free survival rates of 4th-CoCs and matched MoPs were 98.5% and 98.2% (*P* = 0.24), with no significant difference in revision risk found in this group (HR 0.86, 95% CI 0.53–1.38).

**Table 2:** Adjusted hazard ratio (HR) with confidence intervals (CIs) of first adverse clinical outcomes after adjustment of major covariates based on proportional hazard model

	3rd-CoCs group (Ref: MoPs)		4th-CoCs group (Ref: MoPs)	
	HR (95%CI)^c^	*P* value	HR (95%CI)^c^	*P* value
Revision	0.53 (0.34–0.85)^*^	0.01	0.86 (0.53–1.38)	0.53
Postoperative complications ^a^	0.69 (0.49–0.99)^*^	0.04	0.76 (0.54–1.08)	0.12
medical complications ^b^	0.63 (0.28–1.40)	0.26	0.29 (0.15–0.54)^**^	< 0.01

MoPs indicate metal-on-polyethylene implants; CoCs, ceramic-on-ceramic implants

* P value < 0.05 ** P value < 0.01

a. Postoperative complications: revision, periprosthetic joint infection, dislocation, periprosthetic fracture

b. 90-day medical complications: pulmonary embolism, pneumonia, deep vein thrombosis, or sepsis

c. Adjusting covariates: Age group, Insured status, Elixhauser comorbidity indices, Hospital ownership, Healthcare facility level, Hospital region, Bone cement, Modular neck stem, years of Specialty Practice, and surgeon’s surgical volume in the year before surgery

**Fig. 2:**
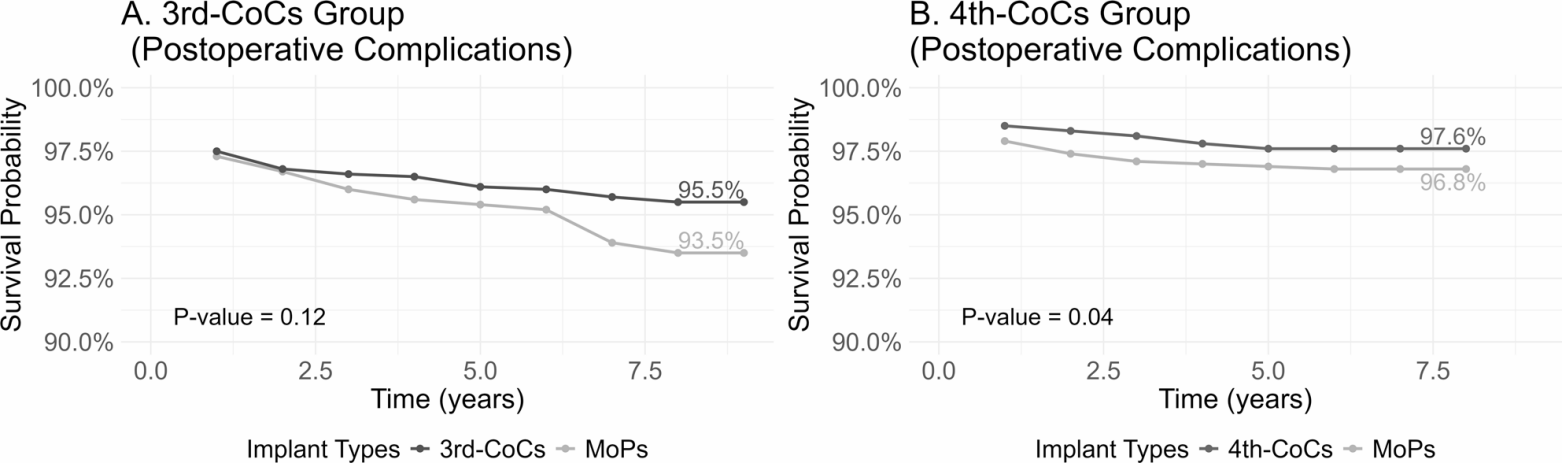
Postoperative complication-free survival rates between two generations of CoCs and matched MoPs. Notes: MoPs indicate metal-on-polyethylene implants; CoCs, ceramic-on-ceramic implants. The y-axis started at 90% to provide a detailed view of the high survival probability range

The postoperative complication-free survival rates for 3rd-CoCs and matched MoPs were 95.6% and 93.5%, respectively (*P* = 0.12; Fig. 2). After adjusting for confounding factors, 3rd-CoCs had a protective effect (HR 0.69, 95% CI 0.49–0.99; Table 2). The postoperative complication-free survival rate for 4th-CoCs and matched MoPs were 97.7% and 96.8%, respectively (*P* = 0.04; Fig. 2), with no significant difference in risk observed for 4th-CoCs (HR 0.76, 95% CI 0.54–1.08; Table 2).

**Fig. 3:**
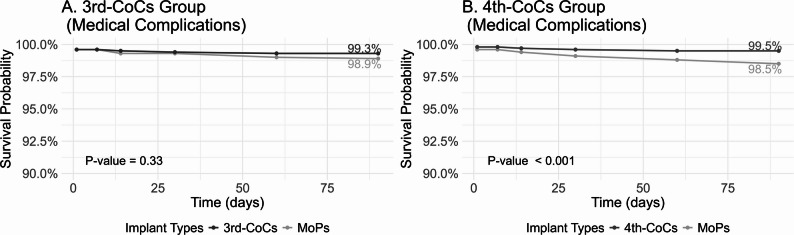
Medical complication-free survival rates between two generations of CoCs and matched MoPs. Notes: MoPs indicate metal-on-polyethylene implants; CoCs, ceramic-on-ceramic implants. The y-axis started at 90% to provide a detailed view of the high survival probability range

The 90-day medical complication-free survival rate for 3rd-CoCs and matched MoPs were 99.3% and 98.9%, respectively (*P* = 0.33; Fig. 3), with no significant difference in risk (HR 0.63, 95% CI 0.28–1.40; Table 2). By comparison, the 90-day medical complication-free survival rate for 4th-CoCs and matched MoPs were 99.5% and 98.5% (*P* < 0.01; Fig. 3), with an adjusted risk of 0.29 for 4th-CoCs (95% CI 0.15–0.54; Table 2).

The incremental differences in event-free survival prediction for the 3rd-CoCs group were 1.21 for revision, 1.09 for postoperative complications, and 0.33 for medical complications. In the 4th-CoCs group, the predictions were 0.33 for revision, 0.78 for postoperative complications, and 0.96 for medical complications (Supplementary Table 3).

### Predicted and incremental healthcare costs by observation period

Because the outcomes of revision- and postoperative complication-free survival were followed over the full study period while the outcome of medical complication-free survival was observed for 90 days, we estimated two sets of healthcare costs corresponding to those two observation periods. For 3rd-CoCs, the overall predicted healthcare costs were US$14,085 (95% CI 13,874–14,295), and the 90-day predicted costs were US$5,592 (95% CI 5,566–5,619). Compared with the MoPs, the incremental healthcare costs for 3rd-CoCs were US$846 over the entire observation period and US$1,404 within the 90-day period.

Similarly, for 4th-CoCs, the overall predicted healthcare costs were US$11,870 (95% CI 11,742–11,998), and the 90-day predicted costs were US$7,026 (95% CI 7,008–7,045). Compared with the MoPs, the incremental healthcare costs for 4th-CoCs were US$2,483 over the entire observation period and US$2,814 within the 90-day period (Supplementary Table 3).

### ICER results and planes for CoCs and MoPs

**Fig. 4:**
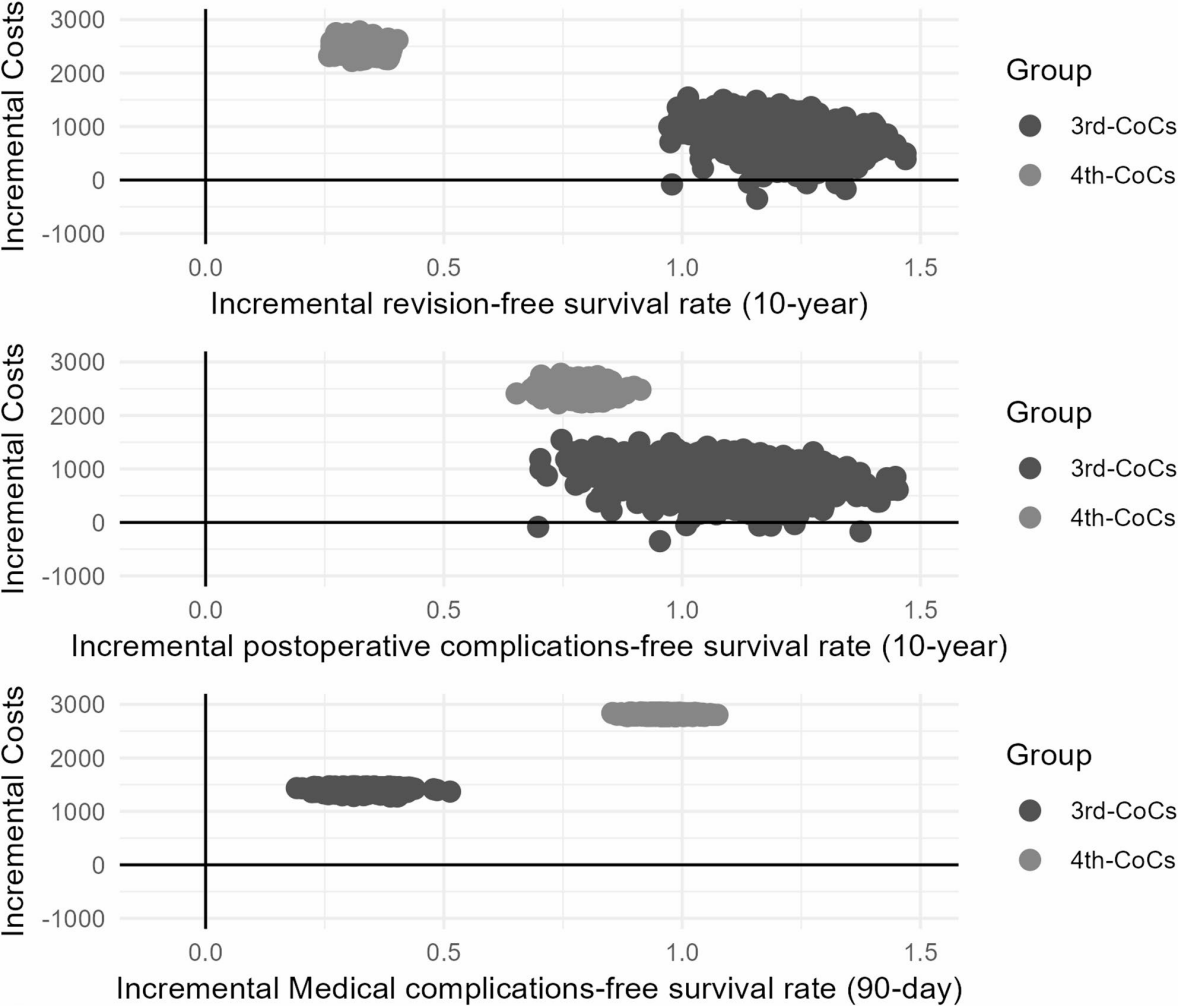
ICER scatterplots show healthcare cost differences versus differences in three effective outcome. Notes: MoPs indicate metal-on-polyethylene implants; CoCs, ceramic-on-ceramic implants. Scatterplots showing cost-effectiveness profiles obtained by comparisonwith the well-matched 1:1 group. Individuals using 3rd- and 4th-CoCs were comparedwith those using MoPs. The dot in ICER scatterplots represents each of the 1,000bootstrapped replications

The ICER results (Supplementary Table 3) revealed that the ratios for the 3rd-CoCs group were 704.01 (95% CI 688.46–719.55) for revision and 794.10 (95% CI 775.21–812.98) for postoperative complications, whereas the ratios for the 4th-CoCs group were 7,605.00 (95% CI 7,566.24–7,643.76) and 3,188.39 (95% CI 3,176.69–3,200.09), respectively, over the entire study period. Conversely, the ICERs for medical complications were 4,375.88 (95% CI 4,338.56–4,413.20) for the 3rd-CoCs group and 2,947.12 (95% CI 2,940.42–2,953.81) for the 4th-CoCs group within 90 days. The ICER plane (Fig. 4) showed that almost all scatterplots fell within the first quadrant, indicating positive incremental healthcare costs and event-free survival, suggesting that both CoCs incurred greater incremental costs and achieved higher incremental event-free survival than MoPs.

## Discussion

This nationwide matched retrospective cohort study evaluated clinical outcomes and cost-effectiveness between 3rd- and 4th-CoCs in THA. Compared with patients using MoPs, those with 3rd-CoCs were at significantly lower risks of revision and postoperative complications: with ICERs of approximately US$704 per 1% improvement in revision-free survival and US$794 per 1% improvement in postoperative complication-free survival. Although patients using 4th-CoCs were at slightly higher crude survival rates, the benefit was limited to reducing 90-day medical complications, and their ICERs exceeded US$2,900 per 1% improvement in medical complication-free survival.

### Revision and complications risks compared across CoCs and MoPs studies

Previous registry-based studies have reported inconsistent evidences on whether CoCs reduce long-term revision risk compared with MoPs. For example, the Danish Hip Arthroplasty Registry (2002–2009) reported an 8.7-year cumulative revision incidence of 5.4% for CoCs versus 5.3% for MoPs [27]. Similarly, the Netherlands Joint Replacement Registry (2007–2016) found 9-year revision incidence of 4.1% for CoCs and 3.9% for MoPs and a lower revision risk for CoCs compared with MoPs (HR 0.82, 95% CI 0.71–0.94; *P* < 0.05) [28]. A network meta-analysis and data from the Australian registry also failed to demonstrate a consistent superiority of CoCs over MoPs in revision outcomes [29, 30]. Such discrepancies likely reflect differences in study periods, implants involvements, and patient characteristics. In our cohort (2009–2019), matched analyses demonstrated lower revision incidence for both CoC generations than in earlier reports: 2.7% vs. 4.4% for 3rd-CoCs vs. MoPs, and 1.5% vs. 1.8% for 4th-CoC vs. MoPs. While crude revision rates appeared lower for 4th-CoCs, only 3rd-CoCs were associated with a statistically significant reduction in revision risk after multivariable adjustment. This downward trend in revision rates over calendar time likely results from a combination of factors, including improved implant materials, advancements in surgical technique, and the selection of lower-risk patient groups. Our findings thus complement earlier literature by providing real-world evidence from a more recent procedural era and a rigorously matched analytic design.

We found that 3rd-CoCs were associated with fewer postoperative complications, whereas 4th-CoCs were linked to reduced 90-day medical complications. Although registry studies from Denmark and the US did not identify clear benefits of CoCs in preventing infection or mechanical complications [27, 31], a British registry study reported a 25% reduction in infection-related revisions among CoC users, suggesting a potential advantage related to ceramic surface properties [4]. Furthermore, although some studies have investigated outcomes such as pneumonia, pulmonary embolism and deep vein thrombosis, they did not specifically evaluate implant material as an exposure variable [25, 32, 33]. A recent health policy analysis found that lower OOP payments were associated with prolonged LoS for hip replacement procedures; the authors proposed several contributing factors, including patients’ health status, insurance coverage, surgeon efficiency, use of advanced technologies, and operating room equipment [14]. Therefore, we cautiously interpret the association between 4th-CoCs and reduced 90-day medical complications as potentially influenced, at least in part, by unmeasured confounding factors. The association between 3rd-CoCs and fewer postoperative complications may be related to a lower incidence of periprosthetic joint infections. In contrast, the observed link between 4th-CoCs and reduced 90-day medical complications lacks clinical evidence to explain a plausible mechanism.

### Cost-effectiveness analysis of 3rd- and 4th-CoCs compared with MoPs

While our ICERs were calculated using event-free survival rather than QALYs, prior modeling studies provided useful benchmarks. Considering that the WTP threshold is typically set at US$50,000 per QALY gained in the US and £20,000 per QALY in the UK [34], our ICERs are expressed as costs per incremental event-free survival; thus, there is no standard WTP threshold to directly evaluate our results. An expected-value decision-analysis model estimated that an implant would be considered cost-effective if it reduced the 20-year revision risk by 1.9%–5.6% at an incremental cost of US$1,000–US$3,000 [12]. In our study, the 3rd-CoCs demonstrated a 1.21% improvement in revision-free survival with an incremental cost of US$1,348, which approaches the lower bound of this benchmark. In contrast, the 4th-CoCs showed only a 0.33% gain at a higher cost of US$3,196, suggesting limited short-term value. Additionally, we estimated ICERs separately for postoperative complications and 90-day medical complications; however, due to the lack of comparable benchmarks in the existing literature, these could be considered as exploratory findings.

Follow-up duration has a substantial impact on ICER values. A real-word data study demonstrated that THA becomes increasingly cost-effective over time, with ICERs decreasing as the follow-up period lengthens and achieving high cost-effectiveness after three years [35]. In our study, the maximum follow-up period was 10 years, and the median follow-up durations were 6.2 years for 3rd-CoCs and 3.3 years for 4th-CoCs. Previous registry-based studies have also reported discrepancies between the study period and the actual follow-up durations observed. For example, a Danish study spanning 8.7 years reported a median follow-up duration of 5.0 years for CoCs and 3.9 years for MoPs [36]. In another Dutch study, a 9-year study period yielded a mean follow-up duration of 3.9 years for CoCs [28]. The median duration for CoCs in our study surpassed the 3-year threshold, reflecting cost-effectiveness comparable to that reported in the literature. We disclosed various time horizons to promote transparency and support stakeholders to interpret the short- to mid-term cost-effectiveness evidence in real-world settings. Nevertheless, as the 4th-CoCs in our study had markedly shorter follow-up periods compared with the 3rd-CoCs, due to their later availability, further research using updated NHI data is warranted to better capture the long-term outcomes of 4th-CoCs.

### Strengths and limitations

This study leveraged Taiwan’s NHI claims system, which requires reporting for all THA procedures, including fully covered and copay implants. This structure minimizes selection bias commonly observed in commercial claims datasets and ensures accurate classification of implants, thereby addressing limitations of ICD-based administrative data [37]. By combining exact matching with propensity score matching and adjusting for multiple covariates, we intended to establish a comparable cohort of implants and reduce confounding. Furthermore, the inclusion of data on the device copayments enabled a more comprehensive calculation of healthcare expenditures.

Several other limitations need to be reflected. First, our dataset did not include information on professional caregiver fees, daily activity levels, surgical approach, or prosthetic head size and these factors likely contributed to residual confounding. For instance, the ICERs reported in our study may represent a lower-bound estimate, without accounting for the out-of-pocket payments such as professional caregiver fees. Moreover, the absence of clinical details like surgical approach or prosthetic head size in the NHI database could have introduced bias when comparing the effectiveness of CoCs versus MoPs. Second, in order to ensure comparable study cohorts, we excluded individuals with cancer or bilateral THA to construct the comparison group of MoP users, with baseline characteristics similar to the CoCs groups with the five major comorbid conditions. However, the unmatched rates were 4.3% in the 3rd-CoC group and a higher rate of 14.8% in cases with 4th-CoC. Most unmatched 4th-CoC cases, receiving surgery in 2016–2019, had comorbidities associated with higher mortality, and therefore had shorter follow-up periods. Excluding these patients from our analytic sample may have led to an underestimation of ICERs in real-world settings, as they would likely generate higher healthcare costs but experience less long-term effectiveness. These exclusions may also limit the generalizability of our findings to high-risk populations.

Third, because quality-of-life (QoL) data were unavailable in the NHI database, we used adverse outcomes as surrogate measures of effectiveness, precluding direct comparison with QALY-based thresholds. Since patient-reported outcomes such as QoL are important for capturing the full benefit of THA, we recommend future studies to collect QoL data among OA patients in Taiwan to facilitate international comparisons. Fourth, although OOP costs before 2015 were estimated using stratified cost-sharing ratios derived from 2015 to 2018 data, this imputation method may introduce uncertainty. While formal sensitivity analyses were not feasible due to data limitations, our imputation strategy incorporated implant type, hospital level, and regional variation to mitigate potential bias. Fifth, while the 1,000 bootstrap iterations adopted in this study to derive confidence intervals for the ICER estimates were lower than the recommended practice of 2000–5000 iterations [38], the ICER scatterplots in our study showed tightly clustered distributions, supporting the stability of the bootstrap estimates. Further studies may also consider 2,000 or more iterations to further strengthen the reliability of the confidence interval estimation. Lastly, while the non-parametric bootstrap approach adopted in this study effectively captured real-world variability without imposing model-based assumptions and sufficiently addressed statistical uncertainty, structural uncertainty was not formally evaluated through scenario analyses. Incorporating structured scenario analyses in future research would improve the applicability and comprehensiveness of real-world cost-effectiveness evaluations.

## Conclusions

In countries where MoPs are fully covered by health insurance and copays for 3rd- and 4th-CoCs vary, the ICERs presented in this study provide valuable information for healthcare payers and patients. For patients aged 50 years and older undergoing primary THA for osteoarthritis, 3rd-CoCs appear to be a cost-effective option. However, the cost-effectiveness of 4th-CoCs remains uncertain due to limited long-term data and variability in healthcare system willingness-to-pay thresholds. Payers should consider these findings and seek extended follow-up studies to better assess the cost-effectiveness of newer CoC implants.

## Supplementary Information

Below is the link to the electronic supplementary material.

Supplementary Material 1

## Data Availability

The data that support the findings of this study are available from the Taiwan’s HWDC, but restrictions apply to the availability of these data, which were applied to be used exclusively for the current study, and so are not publicly available.

## Abbreviations

CoCs: Ceramic-on-ceramic implants
3rd-CoCs: Third-generation CoC implants
4th-CoCs: Fourth-generation CoC implants
MoPs: Metal-on-polyethylene implants
THA: Total hip arthroplasty
OA: Osteoarthritis
ICD-9-CM/PCS or ICD-10-CM/PCS: International classification of diseases, ninth or tenth revision, clinical modification/procedure coding system
NHI: National health insurance
OOP: Out-of-pocket expenses
WTP: Willingness-to-pay threshold
ICER: Incremental cost‒effectiveness ratio
QALYs: Quality-adjusted life years
LoS: Length of stay
HR: Hazard ratio
GB: Global budget
CI: Confidence interval
SMD: Standardized mean difference
GLM: Generalized linear model
QoL: Quality-of-life
