# Supplementary material for: The cost-effectiveness of different generations ceramic-on-ceramic implants in primary total hip arthroplasty: a matched population-based study
Source: BMC Health Serv Res. 2025 Dec 9;26:62. doi: 10.1186/s12913-025-13792-5 (PMC12801667; doi:10.1186/s12913-025-13792-5)
Supplement: Supplementary file 1 — Supplementary Material 1 [file 12913_2025_13792_MOESM1_ESM.docx]

**Supplementary Figure 1.** Trends in the choices of patients undergoing THA.

**
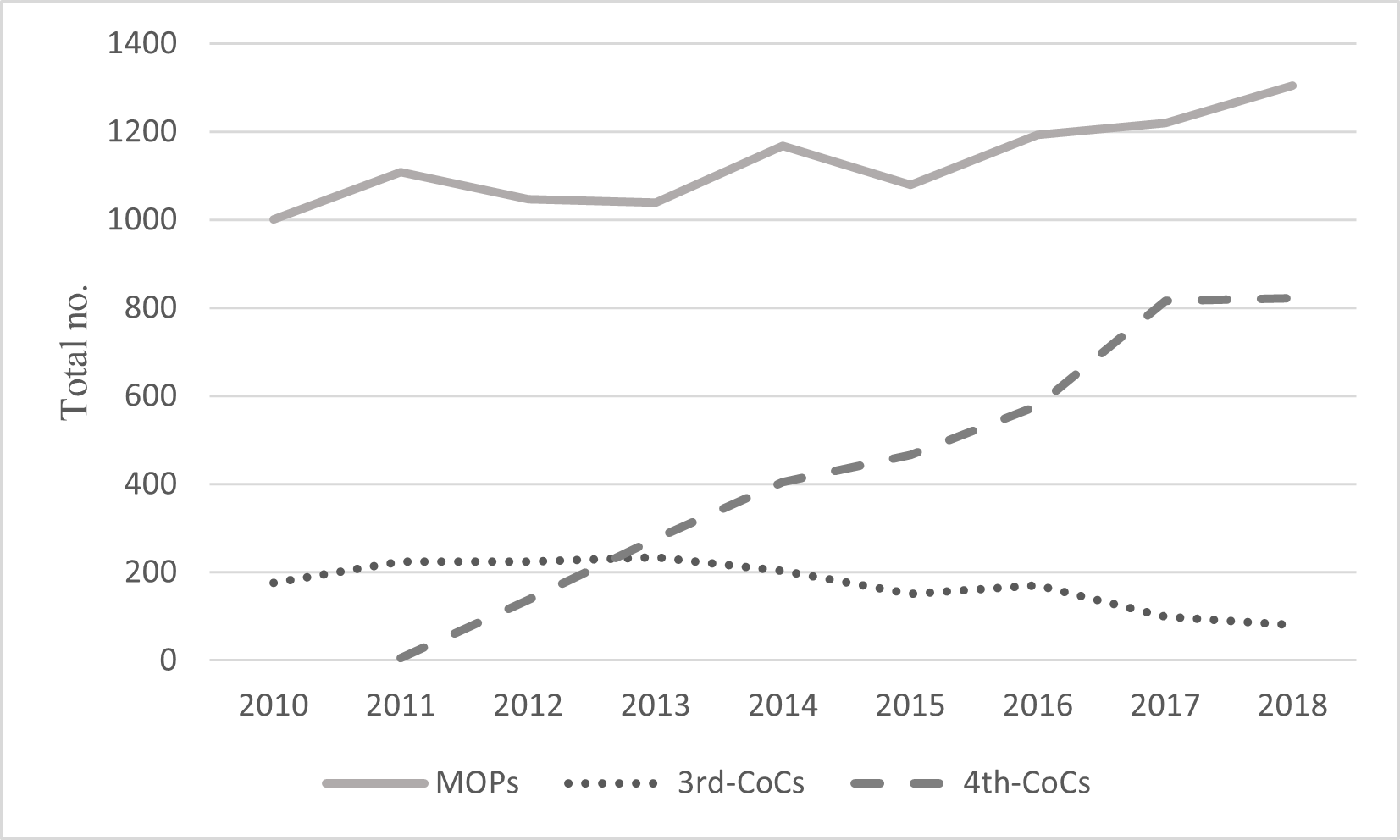
**

THA indicates total hip arthroplasty; MoPs, metal-on-polyethylene implants; CoCs, ceramic-on-ceramic implants.

**Supplementary Figure 2.** Flow diagram outlining how the study cohorts were established.


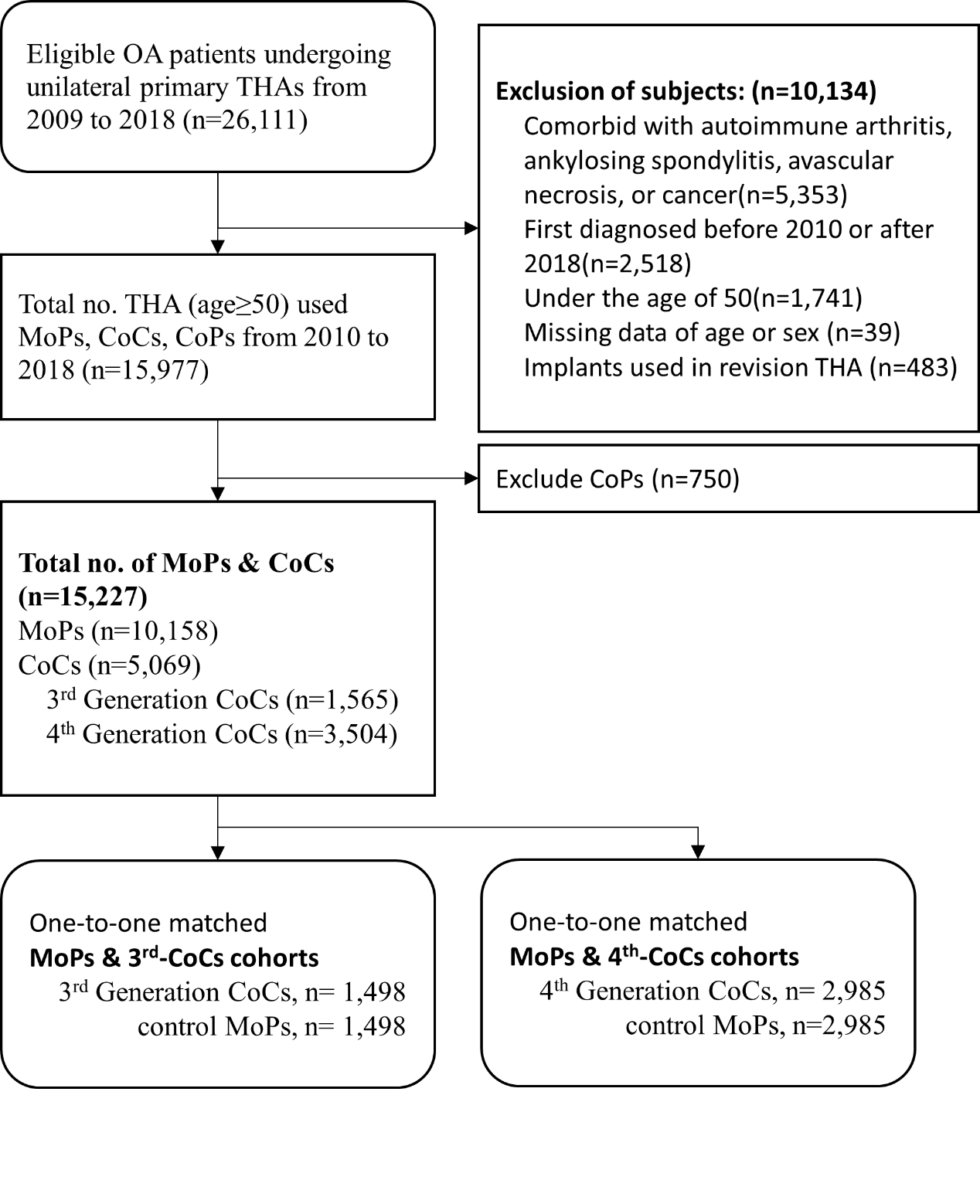


OA indicates osteoarthritis; THA, total hip arthroplasty; MoPs, metal-on-polyethylene implants; CoCs, ceramic-on-ceramic implants; CoPs, ceramic-on-polyethylene implants.

One-to-one matching including age, sex, procedure year, acute myocardial infarction, stroke, chronic obstructive pulmonary disease, end-stage renal disease, and cirrhosis and propensity scores within a ±0.2 range.

Detailed covariate balance checks are presented in Table 1.

**Supplementary Table 1.** Detailed codes of International Classification of Diseases - Clinical Modification (ICD-CM) or Procedure Coding System (PCS) and those of National Health Insurance (NHI).

| Category |  | ICD-CM or PCS | |  |  |  |  | NHI codes |
| --- | --- | --- | --- | --- | --- | --- | --- | --- |
|  |  | ICD-9 |  |  | ICD-10 |  |  |  |
|  |  | CM | PCS |  | CM | PCS |  |  |
| Patients Identification |  |  |  |  |  |  |  |  |
| Osteoarthritis |  | 715.35 |  |  | M16 |  |  |  |
| Hip arthroplasty |  |  | 81.51 |  |  | 0SRB, 0SR9 |  |  |
| Clinical outcomes |  |  |  |  |  |  |  |  |
| Revision |  |  |  |  |  |  |  | 64201 |
| Postoperative complications |  |  |  |  |  |  |  |  |
| 1. Revision |  |  |  |  |  |  |  | 64201 |
| 1. Infection |  | 730, 996.66, 909.3, 998.51, 998.59 |  |  | M86, M89.5, M90.5, T84.51, T84.52, T81.4 |  |  | 48004-48006, 64052 |
| 1. Dislocation |  |  |  |  |  |  |  | 64064, 64073 |
| 1. Fracture |  | 996.44 |  |  | T84.04, T84.1, T84.2-T84.4, T84.8, T84.9 |  |  | 64028 |
| Medical complications |  |  |  |  |  |  |  |  |
| 1. Pulmonary embolism |  | 415.1 |  |  | I26.9, T80.0, T81.7, T82.8 |  |  |  |
| 1. Deep vein thrombosis |  | 451.1, 451.2, 451.8, 451.9, 453.4, 453.8, 453.9 |  |  | I80.1-I80.3, I80.9, I82.2, I82.4- I82.9, I82.A- I82.C |  |  |  |
| 1. Pneumonia |  | 480-486 |  |  | J12, J13, J15-J18, A22, A37, A48, B25, B44 |  |  |  |
| 1. Sepsis |  | 995.9 |  |  | A41.9 |  |  |  |

**Supplementary Table 2.** National Health Insurance (NHI) codes of the implants.

| Implants | Brand | NHI codes |
| --- | --- | --- |
| MoPs |  | FBHPA |
| 3^rd^-CoCs (Alumina, or Zirconia) | Microport | FBHPCCERA45D, FBHPCCERA15D |
|  | Stryker | FBHPCCERA1S2 |
|  | United | FBHPCCERA1U0 |
|  | Wright | FBHPCCERA1W2, FBHPCCERA4W2 |
|  | Zimmer | FBHPCCERA1Z1 |
| 4^th^-CoCs (Composite-CoC, include Zirconia-toughened alumina matrix composite, or Oxidized Zirconium) | Microport | FBHPCCERAH5D, FBHPCCERAP5D |
|  | United | FBHPC7100NU0, FBHPCUTF1TU0 |
|  | Wright | FBHPCCERA7W2 |
|  | Zimmer | FBHPCCERA3Z1, FBHPCCERA4Z1 |
|  | Depuy | FBHPCCERA1DP |

| **Supplementary Table 3.** Incremental cost-effectiveness ratio (ICER) of CoCs compared with MoPs. | | | | | | |
| --- | --- | --- | --- | --- | --- | --- |
| Outcomes | Implant group | Healthcare costs^c^ | Event-free survival^d^ | Incremental difference | | ICER |
|  |  | Mean (95% CI) | Mean  (%, 95% CI) | Healthcare costs | Event-free |  |
|  |  |  |  |  | survival (%) |  |
| 3^rd^-CoCs matched group (1498 pairs) | | | | | | |
| Revision^a^ | MoPs | 13238  (12741 – 13736) | 96.76  (96.62 - 96.89) | - | - | - |
|  | 3rd-CoCs | 14085  (13874 – 14295) | 97.97  (97.88 - 98.06) | 846 | 1.21 | 704.01  (688.46 - 719.55) |
| Postoperative complications^a^ | MoPs | 13238  (12,741 – 13736) | 95.06  (94.88 - 95.23) | - | - | - |
|  | 3rd-CoCs | 14085  (13,874 – 14295) | 96.15  (95.98 - 96.32) | 846 | 1.09 | 794.10  (775.21 - 812.98) |
| Medical complications^b^ | MoPs | 4188  (4136 – 4240) | 98.95  (98.88 - 99.01) | - | - | - |
|  | 3rd-CoCs | 5592  (5566 – 5619) | 99.27  (99.23 - 99.32) | 1404 | 0.33 | 4375.88  (4338.56 – 4413.20) |
| 4th-CoCs matched group (2,985 pairs) | | | | | | |
| Revision^a^ | MoPs | 9386  (9263 – 9510) | 98.58  (98.54 - 98.61) | - | - | - |
|  | 4th-CoCs | 11870  (11742 – 11998) | 98.90  (98.88 - 98.93) | 2483 | 0.33 | 7605.00  (7566.24 – 7643.76) |
| Postoperative complications^a^ | MoPs | 9386  (9263 – 9510) | 97.21  (97.15 - 97.26) | - | - | - |
|  | 4th-CoCs | 11870  (11742 – 11998) | 97.99  (97.94 - 98.03) | 2483 | 0.78 | 3188.39  (3176.69 – 3200.09) |
| Medical complications^b^ | MoPs | 4212  (4195 – 4229) | 98.55  (98.49 - 98.61) | - | - | - |
|  | 4th-CoCs | 7026  (7008 – 7045) | 99.51  (99.47 - 99.54) | 2814 | 0.96 | 2947.12  (2940.42 - 2953.81) |
| MoPs indicate metal-on-polyethylene implants; CoCs, ceramic-on-ceramic implants. | | | | | | |
| a. The event-free survival rate was observed over the full study periods. | | | | | | |
| b. The event-free survival rate was observed over 90 days.  c. Healthcare costs in the predicted generalized linear model in gamma log-link (adjusted for implant group, insured status, Elixhauser comorbidity indices, hospital ownership, healthcare facility level, hospital region, comorbidities, years of specialty practice, surgeon's surgical volume in the year before surgery, modular neck stem, bone cement, and follow-up duration).  d. Event-free survival in the predicted Cox model (adjusted for age group, insured status, Elixhauser comorbidity indices, hospital ownership, healthcare facility level, hospital region, bone cement, modular neck stem, years of specialty practice, and surgeon's surgical volume in the year before surgery). | | | | | | |

| **Supplementary Table 3.** Incremental cost-effectiveness ratio (ICER) of CoCs compared with MoPs. | | | | |  |  |
| --- | --- | --- | --- | --- | --- | --- |
| Outcomes | Implant group | Healthcare costs^c^ | Event-free survival^d^ | Incremental difference | | ICER |
|  |  | Mean (95% CI) | Mean  (%, 95% CI) | Healthcare costs | Event-free |  |
|  |  |  |  |  | survival (%) |  |
| 3^rd^-CoCs matched group (1498 pairs) | | | | | | |
| Revision^a^ | MoPs | 13238  (12741 – 13736) | 96.76  (96.62 - 96.89) | - | - | - |
|  | 3rd-CoCs | 14085  (13874 – 14295) | 97.97  (97.88 - 98.06) | 846 | 1.21 | 704.01  (688.46 - 719.55) |
| Postoperative complications^a^ | MoPs | 13238  (12,741 – 13736) | 95.06  (94.88 - 95.23) | - | - | - |
|  | 3rd-CoCs | 14085  (13,874 – 14295) | 96.15  (95.98 - 96.32) | 846 | 1.09 | 794.10  (775.21 - 812.98) |
| Medical complications^b^ | MoPs | 4188  (4136 – 4240) | 98.95  (98.88 - 99.01) | - | - | - |
|  | 3rd-CoCs | 5592  (5566 – 5619) | 99.27  (99.23 - 99.32) | 1404 | 0.33 | 4375.88  (4338.56 – 4413.20) |
| 4th-CoCs matched group (2,985 pairs) | | | | | | |
| Revision^a^ | MoPs | 9386  (9263 – 9510) | 98.58  (98.54 - 98.61) | - | - | - |
|  | 4th-CoCs | 11870  (11742 – 11998) | 98.90  (98.88 - 98.93) | 2483 | 0.33 | 7605.00  (7566.24 – 7643.76) |
| Postoperative complications^a^ | MoPs | 9386  (9263 – 9510) | 97.21  (97.15 - 97.26) | - | - | - |
|  | 4th-CoCs | 11870  (11742 – 11998) | 97.99  (97.94 - 98.03) | 2483 | 0.78 | 3188.39  (3176.69 – 3200.09) |
| Medical complications^b^ | MoPs | 4212  (4195 – 4229) | 98.55  (98.49 - 98.61) | - | - | - |
|  | 4th-CoCs | 7026  (7008 – 7045) | 99.51  (99.47 - 99.54) | 2814 | 0.96 | 2947.12  (2940.42 - 2953.81) |
| MoPs indicate metal-on-polyethylene implants; CoCs, ceramic-on-ceramic implants. | | | | | | |
| a. The event-free survival rate was observed over the full study periods. | | | | | | |
| b. The event-free survival rate was observed over 90 days.  c. Healthcare costs in the predicted generalized linear model in gamma log-link (adjusted for implant group, insured status, Elixhauser comorbidity indices, hospital ownership, healthcare facility level, hospital region, comorbidities, years of specialty practice, surgeon's surgical volume in the year before surgery, modular neck stem, bone cement, and follow-up duration).  d. Event-free survival in the predicted Cox model (adjusted for age group, insured status, Elixhauser comorbidity indices, hospital ownership, healthcare facility level, hospital region, bone cement, modular neck stem, years of specialty practice, and surgeon's surgical volume in the year before surgery). | | | | | | |
